# DNA Methylation of Postnatal Liver Development in Pigs

**DOI:** 10.3390/genes15081067

**Published:** 2024-08-13

**Authors:** Yuhao Wang, Hongling Jin, Xingyan Tong, Huan Yu, Xuewei Li, Bo Zeng

**Affiliations:** 1 Key Laboratory of Livestock and Poultry Multi-Omics, Ministry of Agriculture and Rural Affairs, Sichuan Agricultural University, Chengdu 611130, China; 18628240370@163.com (Y.W.); 17361123793@163.com (X.T.); 2 College of Animal Science and Technology, Sichuan Agricultural University, Chengdu 611130, China; 18716160560@163.com (H.J.); 19162893316@163.com (H.Y.); 3 Key Laboratory of Agricultural Bioinformatics, Ministry of Education, Sichuan Agricultural University, Chengdu 611130, China

**Keywords:** pig, liver, DNA methylation, postnatal development, 5-methylcytosine

## Abstract

DNA methylation plays an important role in the development and tissue differentiation of eukaryotes. In this study, bisulfite sequencing (BS-seq) technology was used to analyze the DNA methylation profiles of liver tissues taken from Rongchang pigs at three postnatal feeding stages, including newborn, suckling, and adult. The DNA methylation pattern across the genomes or genic region showed little difference between the three stages. We observed 419 differentially methylated regions (DMRs) in promoters, corresponding to 323 genes between newborn and suckling stages, in addition to 288 DMRs, corresponding to 134 genes, between suckling and adult stages and 351 DMRs, corresponding to 293 genes, between newborn and adult stages. These genes with DMRs were mainly enriched in metabolic, immune-related functional processes. Correlation analysis showed that the methylation level of gene promoters was significantly negatively correlated with gene expression. Further, we found that genes related to nutritional metabolism, e.g., carbohydrate metabolism (FAHD1 and GUSB) or fatty acid metabolism (LPIN1 and ACOX2), lost DNA methylation in their promoter, with mRNA expression increased in newborn pigs compared with those in the suckling stage. A few fatty acid metabolism-related genes (SLC27A5, ACOX2) were hypomethylated and highly expressed in the newborn stage, which might satisfy the nutritional requirements of Rongchang pigs with high neonatal birth rates. In the adult stage, HMGCS2—which is related to fatty acid β-oxidation—was hypomethylated and highly expressed, which explains that the characteristics of high energy utilization in adult Rongchang pigs and their immune-related genes (CD68, STAT2) may be related to the establishment of liver immunity. This study provides a comprehensive analysis of genome-wide DNA methylation patterns in pig liver postnatal development and growth. Our findings will serve as a valuable resource in hepatic metabolic studies and the agricultural food industry.

## 1. Introduction

Physiological processes in multicellular organisms, including normal growth and development, involve finely regulated changes in gene expression. DNA methylation is one of the major epigenetic modification mechanisms involved in eukaryotes and it plays a crucial role in the regulation of gene expression [1]. Studies have shown that the DNA methylation pattern is not static. In the process of development, maturation and aging, the methylation level changes via the interaction of the physiological cycle and the environment [2,3]. DNA methylation usually involves the addition of methyl groups to cytosines at cytosine–guanine (CpG,CG) sites, which often leads to the inactivation of transcription in order to achieve the regulatory purpose of silencing gene expression [4]. The regulatory pattern of DNA methylation across the genome is complex and varies in different gene regions. It is generally believed that DNA methylation in promoter regions is associated with decreased expression [4,5]. However, the correlation between some methylation patterns within genes and gene expression is complex. Therefore, the detection and analysis of global gene methylation levels and patterns in tissues at different growth and development stages are essential to understanding the relationship between tissue spatial–temporal-specific methylation and tissue spatial–temporal-specific gene expression. 

Animals face tremendous environmental changes after birth, which are accompanied by diverse changes in biological functions during growth and development. The liver is the most functional digestive organ in animals and has the largest secretory gland in the body. It directly participates in the metabolism of amino acids, sugars, fats and other substances in the body by secreting bile, enzymes and hormones [6]. After the birth of animals, the material metabolism, development, and function of tissues and organs in the body undergo drastic changes, the liver tissues and organs grow rapidly, and various functions gradually mature. In this process, the function of the liver is also regulated as needed. In the reports on humans and mice, researchers explored the changes in liver methylation at 3~4 different developmental stages [7,8,9], but little is known about the methylation information of suckling piglets and adult pigs. In order to explore the changes in 5-methylcytosine (5mC) methylation during liver development, we generated 5mC methylation profiles at three developmental stages of pigs—newborn (0 day), suckling (21 day), and adult (2 year)—and compared DNA methylation and gene expression in liver tissue. We characterized the developmental transcriptome distribution pattern and analyzed the relationship between gene expression and 5mC modification. We also identified a wide range of 5mC-modified genes, which may contribute to the potential functional differences in liver development stages of the Rongchang pigs. These results provide a resource for the identification of DNA methylation modifications in the liver and extend our knowledge regarding the role of 5mC modifications in mammalian organ development and growth. 

## 2. Materials and Methods

### 2.1. Ethics Statement

All studies involving animals were conducted according to the Regulations for the Administration of Affairs Concerning Experimental Animals (Ministry of Science and Technology, China, revised in March 2017). All experimental procedures and sample collection in this study were approved by the Institutional Animal Care and Use Committee (IACUC) of Sichuan Agricultural University, under permit No. DKY-2018102003. Animals were allowed free access to food and water under normal conditions, and were humanely sacrificed as necessary, to ameliorate suffering.

### 2.2. Animal Experiment and Sample Preparation

Liver tissues were isolated from healthy female Rongchang pigs (Rongchang pig, a Chinese indigenous breed) on postnatal day 0 (Newborn, *n* = 2), on postnatal day 21 (Suckling, *n* = 2), and at the 2-year stage (Adult, *n* = 2) for RNA and DNA isolation, as well as for histological studies. All the pigs used in this research were raised on a Rongchang pig elite reservation farm in Chongqing. The animals had different sources of diets: the newborn piglets received nutrients through the sow placenta, suckling piglets fed on the breast milk of the mother sow, and two-year-old adults fed on balanced artificial diets. Food was withheld from the animals on the night before they were sacrificed. Animals were humanely killed to ameliorate suffering via intravenous injection with 2% pentobarbital sodium (25 mg/kg). The liver of each animal was separated rapidly from each respective carcass, immediately frozen in liquid nitrogen, and stored at −80 °C until use. 

### 2.3. RNA-Seq

All RNA-seq data (liver tissues, *n* = 6) were derived from our previous study and downloaded from a NCBI Gene Expression Omnibus (GEO) with the series accession number GSE87327 [10].

To avoid artificial biases, we removed low-quality reads, including those with ≥10% unidentified nucleotides, >10 nt aligned to the adapter with ≤10% mismatches allowed, and with >50% of bases with a phred quality < 5. The downstream analysis pipeline for RNA-seq data was the same as before. Differentially expressed genes (DEGs) analysis was performed using the edgeR R package (4.4.0); a *p* value < 0.01 and |log2 fold chance (FC)| > 1.0 were set as thresholds to screen out DEGs. 

### 2.4. Library Preparation and Bisulfite Sequencing

For a genome-wide DNA methylation survey, DNA was extracted using the DNeasy Blood & Tissue Kit (Qiagen) and then eluted using 10 mM Tris•Cl, pH 7.5. The quality of the isolated DNA was then measured using a NanoDrop spectrophotometer. The ratio of the absorbance at 260 nm versus 280 nm (A260/A280) provides an estimate of the purity of the DNA. The A260/A280 value should be 1.8 to 2.0 for each DNA sample in order to guarantee quality. The initial volume of DNA for each sample should be at least 5 μg to ensure the success of subsequent uses.

After the samples were qualified for detection, a certain proportion of negative control (lambda DNA) was added. First, the genomic DNA was randomly interrupted at 200–300 bp by Covaris S220. The broken DNA fragment was repaired by end repair, an A-tail was added, and the fragment was ligated with a sequencing adaptor via the methylation modification of all cytosines. This was followed by bisulfite treatment (with EZ DNA Methylation Gold Kit, Zymo Research, Inc., Irvine, CA, USA) and finally PCR amplification to obtain a final DNA library. The library preparations were sequenced on the Illumina NovaSeq 6000 platform in Beijing Glibizzia Bioscience Co., Ltd. (Beijing, China).

After the library was constructed, Qubit2.0 was used for preliminary quantification, and the library was diluted to 1 ng/μL. Then, Agilent 2100 was used to detect the length of the fragment inserted into the library. After meeting the expectations, Q-PCR was used to accurately quantify the effective concentration of the library (the effective concentration of the library was >2 nM) in order to ensure the quality of the library. After qualified library inspection, Illumina HiSeq sequencing is carried out; this occurs after the pooling of different libraries according to the requirements of effective concentration and target data volume. 

### 2.5. Sequencing Read Quality Control and Alignment

FastQC (fastqc_v0.11.5) [11] was used to perform basic statistical analysis on raw data quality. We used trimming to cut off sequencing connectors and low-quality fragments of sequencing data to make more efficient use of sequencing data. The subsequent analysis was based on clean data. We used Trimmomatic software (version 0.36) to achieve raw data Trimming. The data processing steps were as follows: (1) Low-quality reads were truncated using a sliding window with four bases; reads were removed from the window if the average base mass value of the window was lower than 15. Parameter selection relied on SLIDINGWINDOW:4:15. (2) Reads with lead and tail weights lower than 3 or containing N (with N indicating that base information could not be determined) were intercepted. Parameters were selected as follows: LEADING:3 and TRAILING:3. (3) The contaminated reads of the joint were intercepted, and the joint was removed using two modes. 1. The simple alignment mode involved the following: the score showing the alignment between the seed and the joint sequence reached 7 (about 12 bp). 2. The palindrome mode involved the following: when the base score of the overlap area of read 1 and read 2 was greater than 30, the seed part sequence was truncated. Parameter selection was as follows: ILLUMINACLIP: adapter. Fa: 2:30:7:1: true. (4) We discarded reads shorter than 36 nt after pruning. (5) We discarded reads that were not paired. Reads were then aligned, using Bismark (version 0.16.3), to the Sus scrofa 11.1 reference genome (parameters: score_min L, 0, −0.2, −X 700) and annotated with Ensembl GTF data (v109). In order to fully detect the methylation of a genome, the coverage of the genome and the coverage of the C site were statistically analyzed, and the coverage of the C site under each context (that is, the number of reads supporting the context) was calculated.

### 2.6. Detection and Analysis of Methylation Sites

After the comparison results were obtained, Bismark (version 0.16.3) [12] was used for methylation site detection. First, duplicate reads were removed using the deduplicate_bismark script in Bismark_v0.16.3. and we removed the duplicate part of the double-ended sequence. The methylation sites were then detected according to the base type on the C site alignment on the reference genome. The ‘bismark_methylation_extractor --no_overlap’ parameter was used when site calling, and only read 1 information is retained when dealing with the overlap of paired-end reads (PE reads) [10]. A lambda genome was used to calculate the Bisulphite conversion efficiency. Based on the methylation detection results of Bismark, a binomial distribution test B (n, p) was conducted for each C site so as to identify whether this site was a true methylation site. A set of thresholds were established during the analysis in order to find the exact methylation site [13,14]: (1) the sequencing depth was greater than or equal to 5; (2) the q-value was less than or equal to 0.01 [15]. 

For the identified methylation sites, the methylation level was calculated by the formula ML = mC/(mC + umC). Where ML is the methylation level, mC and umC represent the amount of methylated C and unmethylated C, respectively.

Then, we investigated methylation levels in various functional regions of the genome, including promoter (the 2 kb region upstream of the transcription start site (TSS) site), exon, intron, CpG islands (CGI), CGI shore, repeat, etc. We studied the average methylation levels of C sites in various genebodies, both 2 K upstream of TSS and 2 K downstream of the transcription stop site (TES). 

### 2.7. Correlation Analysis and PCA

The correlation of methylation levels between samples is an important index with which to test the reliability of the experiment and whether the sample selection is reasonable. The methylation levels in each bin were calculated using a 2 Kbp/bin sub-sequence environment, and Pearson correlation analysis [16] was performed to test the reliability of the experiment and the reasonableness of sample selection. Principal component analysis (PCA) was performed on the correlation analysis data using the ‘prcomp’ function in the R package (version 4.4.0).

### 2.8. DMR Analysis

The DMRs between different stages were identified using DSS analysis software (version 2.12.0) [17,18,19]. Firstly, the detection of differentially methylated loci (DML) is tested statistically by calling a function, which includes estimating the average methylation level, estimating the dispersion of each CpG site, and conducting a Wald test. In this detection process, DML is tested statistically using smoothing processing. The parameters are set as follows: smoothing.span = 200. Next, it is necessary call DML and set the threshold of the differential methylation detection delta = 0, p.Treshold = 1 × 10^−5^; here, we set delta = 0, meaning that any change in the methylation level will be taken into account, and set the threshold of the *p* value to 1 × 10^−5^. Only when the methylation difference at the CpG site reaches this level of significance is it considered a DML. DMR detection is also based on DML test results in terms of its use of calling functions. Regions with many statistically significant CpG sites were identified as DMRs. Specifically, the minimum length of a DMR is defined as 50 base pairs. A DMR contains at least three CpG sites, and if the distance between two adjacent DMCs is less than or equal to 100 base pairs, they will be combined into one DMR. At least 50% of CpG sites show significant differences in a region that is considered to be a DMR, and the specific parameters are set to minlen = 50, minCG = 3, dis.merge = 100, pct.sig = 0.5. For DMRs, it is necessary to use regional annotations (such as promoters, exons, introns, CGI, CGI shore, repeat, TSS, TES, etc.) and add information with the gene name to the gene region.

### 2.9. Functional Enrichment Analysis for Genes

Gene expression was calculated with featureCounts (version:1.5.0-p3) [20] using input unique alignment reads. Differential expression was determined via DESeq2 (version: 1.12.4) with *p* < 0.05 [21]. Higher-expression genes were defined as genes with FPKM (reads per kilo base of exon model per million mapped reads) log2-transformed fold changes > 1 compared to the other two stages (Student’s *t* test, *p* < 0.05). The Metascape web server “https://metascape.org (accessed on 12 Mar 2024)” was used to perform functional enrichment analysis of Gene Ontology (GO) and Kyoto Encyclopedia of Genes and Genomes (KEGG) pathway categories. Genes with DMRs in their promoters and the DEGs of RNA-seq were mapped onto their respective human orthologs and then submitted to Metascape for enrichment analysis, which included GO biological processes (GO-BPs) and KEGG pathways. Only GO-BP or KEGG pathway terms with q values of less than 0.05 were considered to be significant and included therefore in the list.

## 3. Results

### 3.1. Data Quality of BS-Seq Data

Using BS-seq technology, we obtained DNA methylation data for a total of six samples, including porcine liver tissues at three ages: newborn (0 day), suckling (21 day) and adult (2 year). After the raw sequenced data were sequence-preprocessed (removal of splice sequences, low quality reads), a total of 80–112 G clean bases were generated (Table S1), and the BS conversion rate of all samples exceeded 99%. We compared the paired reads with the Sus scrofa 11.1 reference genome, and found that the alignment rates of BS-seq reads rate were 49.22–66.65% (Table S2), which were similar to the results of previous studies [22,23]. 

### 3.2. Methylation Distribution Characteristics

**Figure 1:**
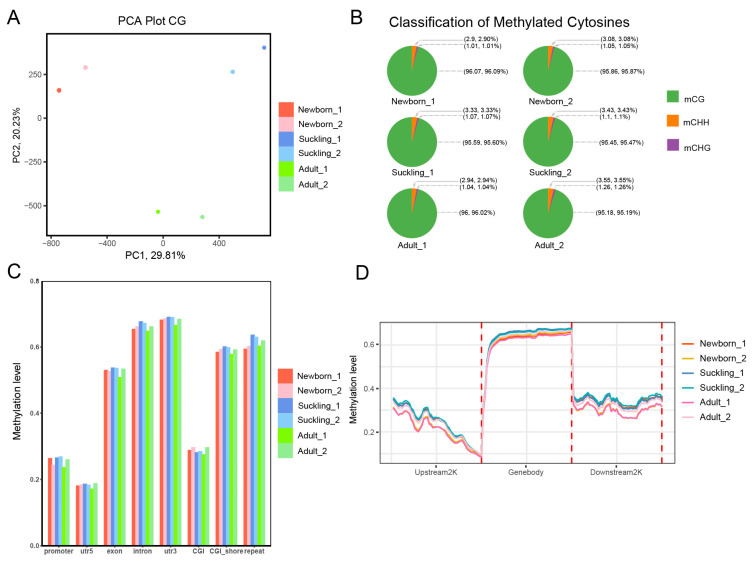
Clustering and PCA analyses of DNA methylation for liver samples. (**A**) PCA of CG methylation levels of all liver samples. Nutritional stage is represented by point shape. Tissues are represented by different colors. (**B**) Percentage of methylcytosines identified for liver samples in context of each sequence. (**C**) Mean CG methylation level in different gene elements, including promoter, utr5′, exon, intron, utr3′, CGI, and CGI_shore repeat in liver. *y* axis represents methylation level. *x* axis shows the different components of genome. (**D**) DNA methylation profile for each gene region is shown by reads that align with unique locus in genome. Methylation pattern diagram of gene regions with upstream 2000 bp and downstream 2000 bp regions in liver. *x* axis indicates position around gene bodies, and *y* axis indicates normalized read number.

Since the data came from different individuals, we performed PCA and Pearson correlation analysis. As shown in Figure 1A, samples from different periods can be distinguished well. Hierarchical cluster analysis showed that the adult and suckling groups were clustered together and separated from the newborn group (Figure S1). These results indicate that the methylation pattern after feeding differs strongly from that of newborns without feeding and that the biological duplication of samples was reasonable.

By classifying the methylation sites, it was found that the methylation of the resulting samples was present in CG, CHG and CHH (H = A, C, or T). More than 80% of cytosine sites in the domestic pig genome are covered by at least 10 individual reads (Table S3). Most of the DNA methylation occurred in CG (CG: 95.19–96.09%, CHG: 1.01–1.26%, CHH: 2.90–3.55%) (Figure 1B).

The methylated cytosines of different gene elements have different functions, and the methylation levels of individual samples in the functional regions of the genes have obvious differences, but the average methylation levels of the same components are similar across individuals (Figure 1C). To investigate genome-wide methylation changes, we assessed the degree of methylation of CG along the gene body upstream and downstream 2 k. Among the selected samples, the overall methylation levels of the samples in different periods were close and the average methylation levels of different elements were similar, with the lowest methylation level seen in the promoter region (Figure 1D). Meanwhile, the analysis along the upstream and downstream of the gene body revealed that the methylation level decreased by 2 kb upstream of the gene start site, while the methylation level was lowest in the region around the gene start site. There was a sharp increase in the gene region, followed by another decrease after the end site of the gene. This methylation pattern is similar to that of other species, such as human [24], mouse [25], panda [26], cow [27], and sheep [28].

### 3.3. Characterization of Porcine Liver Transcriptome

To investigate the expression patterns of DEGs in liver samples at different stages, we first compared the transcriptome characteristics of DEGs pairwise. A total of 3291 DEGs (1684 upregulated and 1607 downregulated) were found in the newborn vs. suckling comparison group; 1898 DEGs (908 upregulated and 990 downregulated) were found in the suckling vs. adult comparison group; and 2310 DEGs (1000 upregulated and 1310 downregulated) were found in the newborn vs. adult comparison group (Figure S2A). Making comparisons with the suckling group, we found that many immune-related GO terms, such as the positive regulation of immune response (G0:0050778), and immune related genes, such as IL18 and STAT3, were highly expressed in the newborn group. In terms of metabolism, the metabolism-related GO terms mainly involve lipid metabolism-related pathways such as the fatty acid metabolic process (GO:0006631), cholesterol metabolism process (GO:0008203), and lipid catabolic process (GO:0016042), and they are represented by the genes LPIN1, SLC27A5, and ACOX2 (Figure S2B, Table S4). Blood vessel development (GO:0001568) and neuron projection development (GO:0031175) illustrate the demand for early neonatal growth. In the suckling group, carbohydrates metabolism-related genes begin to be highly expressed, such as glucose metabolic process (GO:0006006) and pyruvate metabolic process (GO:0006090). The representative genes are IGF2, PGAM1, PGAM2 and PFKM. More biosynthetic and metabolic modalities are beginning to be enhanced, such as ribonucleoprotein complex biogenesis (GO:0022613), peptide metabolic process (GO:0006518), and nucleotide metabolic process (GO:0009117) (Figure S2C, Table S4). This indicates that when pigs enter the suckling stage, with a change of food source, metabolism also undergoes adaptive changes. 

In the comparison between the suckling group and the adult group, the suckling group was more active in glucose metabolism (GO:0005975) (representative genes were PGAM1, PFKM), the peptide metabolic process (GO:0006518) (EEF1A2), and the ribonucleoside triphosphate metabolic process (GO:0009199) (FDFT1, FDXR) than the adult group. The adult group showed more immune-related functions, such as the positive regulation of immune responses (GO:0050778) and a high level of expression of genes including CD86, CD2, and CD8A. Energy metabolism in the adult group showed lipid-dominant characteristics, active pathways related to fatty acid metabolic process (GO:0006631), and a high level of expression of genes related to lipid metabolism such as ACC, D6D, and SCD1 (Figure S2D–G, Table S4).

### 3.4. Methylation Profile of Porcine Liver

#### 3.4.1. Distribution Characteristics of DMRs

**Figure 2:**
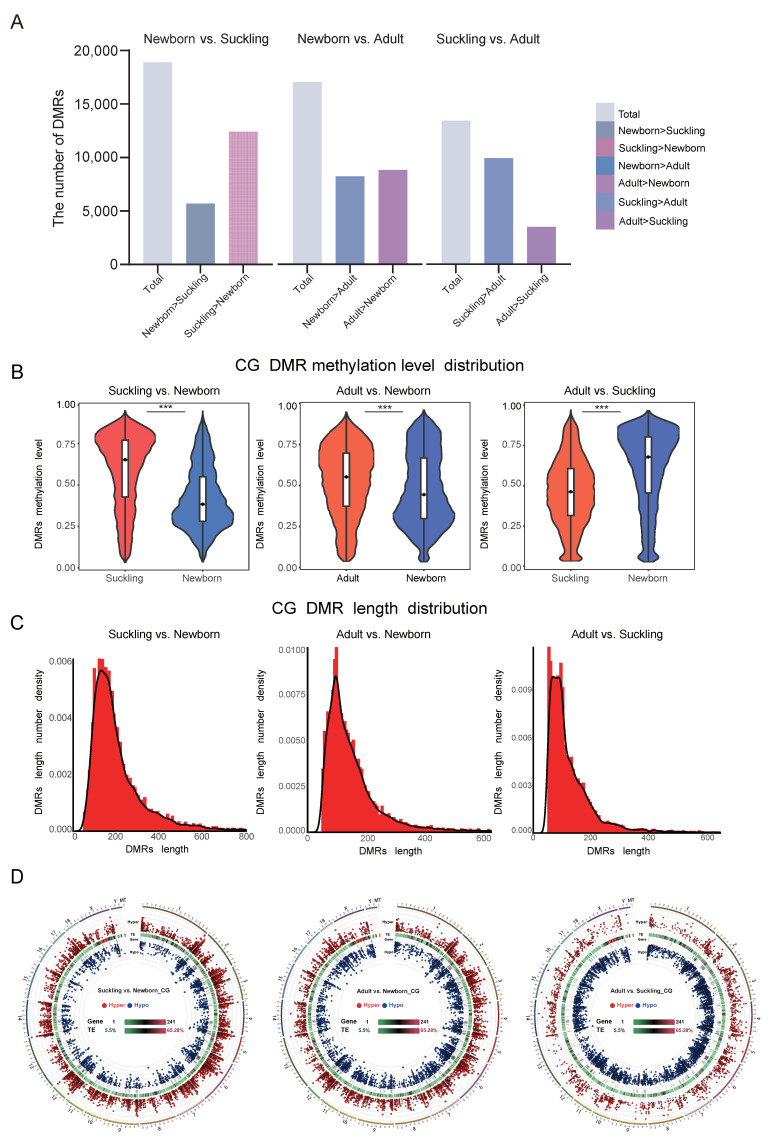
Dynamics of DNA methylation in total genome. (**A**) Bar plot for counts of DMRs and DMR genes between different comparisons in liver. Bar plots for number of total-(grey), hyper- (blue), and hypo- (red) DMRs in liver. (**B**) Distribution of DMR methylation levels across the genome for livers. *x* axis represents comparative combination group and *y* axis represents methylation level. Width of each violin indicates how many points are below this methylation level. Differences were assessed by paired-sample *t*-test and denoted as follows: ***, *p* < 0.001. (**C**) Length distribution of DMRs. *x* axis represents DMR length, *y* axis represents the density value at each length, and black line is distribution fitting curve. (**D**) Circle representation of gene-promoter DMR distribution in chromosomes and its relationship with gene expression. Red circles indicate hyper DMRs, blue circles indicate hypo DMRs, and scale bar from outside to inside represents gene density heatmap and TE component share heatmap, respectively.

We obtained 18,903 DMRs to perform a comparison between the newborn and suckling groups, including 5692 hypermethylated (hyper) and 12,401 hypomethylated (hypo) DMRs; 17,048 DMRs were obtained for comparison between newborn and adult groups (8231 hyper, 8817 hypo), and 13,422 DMRs were obtained for the suckling and adult groups (9922 hyper, 3500 hypo) (Figure 2A).

The distribution of methylation levels in DMRs indicated that the average methylation level of the liver was 0.43 to 0.62 during the neonatal and lactation periods, 0.56 to 0.65 during the adulthood and lactation periods, and 0.49 to 0.60 during neonatal and adulthood periods, with extremely significant differences (*p* < 0.001).

The length distribution of DMRs shows that their characteristics conform to those seen with a normal distribution, and the length of most CpG content in a DMR is less than 500 bp (Figure 2C). DMRs are evenly distributed in the chromosome (Figure 2D).

#### 3.4.2. The DMRs of the Promoter

The relationship between promoter regions and gene silencing has been extensively reported, and it has been shown that DNA methylation in promoter regions is usually associated with transcriptional silencing [29,30,31,32]. Studies have shown extensive changes in DNA methylation in promoter regions during early postnatal development [33,34].

**Figure 3:**
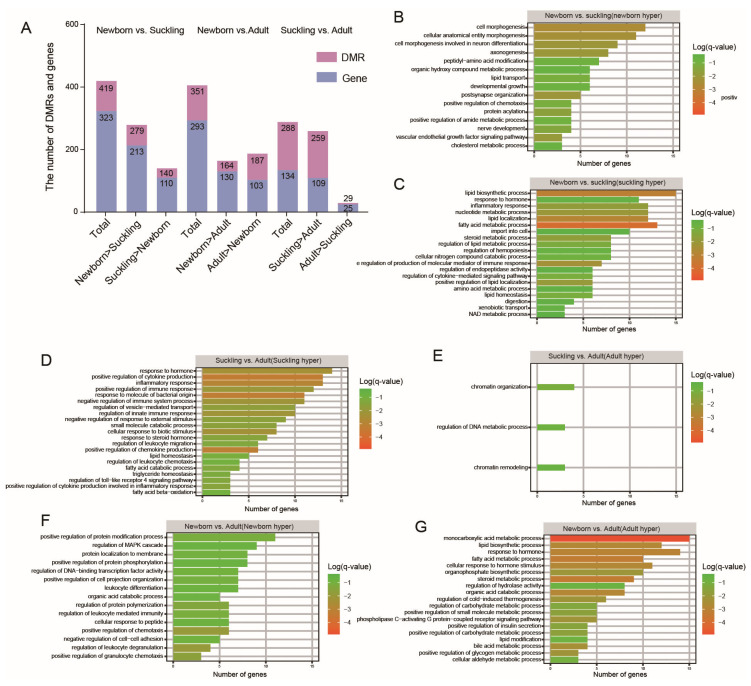
Functional enrichment analysis of differentially methylated promoters between stages. (**A**) Counts of gene-promoter DMRs and related genes between different comparisons in liver. (**B**–**G**) GO terms with gene-promoter differentially methylated genes between stages. Length of bar indicates number of hit genes in each term and color of bar represents q-value.

We observed 850 DMRs (259 hyper, 591 hypo) in the newborn vs. suckling group and 832 DMRs (417 hyper, 414 hypo) in the newborn vs. adult group. There were 728 DMRs (665 hyper, 63 hypo) in the suckling vs. adult group (Table S5). We further identified DMRs with negative correlations between methylation and expression. There were 419 DMRs related to 323 genes in the newborn vs. suckling group (279 hyper DMRs related to 213 genes, 140 hypo DMRs related to 110 genes). The newborn vs. adult group had 351 DMRs related to 293 genes (164 hyper DMRs related to 130 genes, 187 hypo DMRs related to 103 genes). There were 288 DMRs related to 134 genes in the suckling vs. adult group (259 hyper DMRs related to 109 genes, 29 hypo DMRs related to 25 genes) (Figure 3A, Table S5).

We further performed functional enrichment analysis for genes whose expression was negatively correlated with DMRs (Figure 3B–G and Figure S3, Table S6). In the newborn vs. suckling group, we found that the hypermethylated genes in the suckling group were involved in immune responses (Figure S4), such as the positive regulation of the production of molecular mediators of immune responses (GO:0002702); fatty acid metabolism (Figure S4), such as fatty acid metabolism process (GO:0006631); PPAR signaling pathways (hsa03320); the regulation of lipid metabolic process (GO:0019216); and the positive regulation of lipid localization (GO:1905954). They are involved in the metabolism of other substances, such as in the nucleotide metabolism process (GO:0009117) and steroid metabolic process (GO:0008202). In the newborn group, hypermethylated genes were associated with growth and development, such as cell morphogenesis (GO:0000902), involving the Ras signaling pathway (hsa04014) (Figure 3B).

In the newborn vs. adult group, the enrichment results of hypermethylated genes in the adult group were related to steroid and lipid synthesis and catabolism, such as the steroid metabolic process (GO:0008202); fatty acid oxidation (GO:0019395); the bile acid metabolic process (GO:0008206); and carbohydrate metabolism, such as the regulation of the carbohydrate metabolic process (GO:0006109). The hypermethylated genes in the newborn group were dominated by immune concerns such as the regulation of leukocyte-mediated immunity (GO:0002703). The metabolic process is mainly related to protein processing, such as the positive regulation of the protein modification process (GO:0031401) (Figure 3G). 

In contrast, the differences between the adult and suckling groups are mainly related to the highly methylated genes in the suckling group, including immune and inflammation-related genes such as those involved in the negative regulation of the immune system process (GO:0002683) and the positive regulation of cytokine production, which is involved in inflammatory responses (GO:1900017). The metabolic process involves fatty acid β-oxidation (GO:0006635) and lipid homeostasis (GO:0055088) (Figure 3D).

#### 3.4.3. Genes with Differential Expression in the Promoter DMR

We assessed gene-promoter differentially methylated DEGs (*p* < 0.01) with a negative correlation between methylation and expression. A total of 99 genes with differential methylation levels and expression were identified between the newborn and suckling groups, 80 differential genes were found between the newborn and adult groups, and 48 differential genes were found between the suckling and adult groups (Table S7).

Compared to the newborn group, the suckling and adult groups had 13 shared hypermethylated genes (Table S8). According to the functional enrichment results, they were involved in drug metabolism (CYP2A13, CYP2B6), carbohydrate metabolism (FAHD1), fatty acid metabolism (SLC44A3, SLC27A5), lipids metabolism (IDI1, HSD3B1), and small-molecule transport (ANO1, LCN12). Three genes (CLSPN, PGF, ZNF532), hypermethylated in newborns and negatively correlated to gene expression, were associated with the cell cycle and developmental process (CLSPN, PGF, ZNF532). Additionally, we found that 4 genes were hypermethylated and significantly underexpressed in the suckling group relative to both newborn and adult stages. The distribution was immune-related (STAT2, RFX5) and related to fatty acid metabolism (ACSM2A, PRAP1).

**Figure 4:**
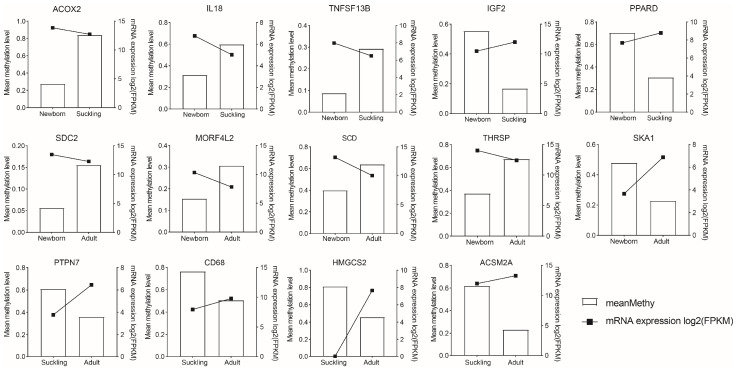
The expression levels (line graph) and methylation degree (bar graph) in promoters for partial genes of comparison groups. Genes (top 14) were screened according to their methylation level and gene expression difference. The right *y* axis indicates the gene expression data which were displayed in a line chart (log2 FPKM), and the left *y* axis indicates the average methylation level of genes, shown as histogram.

In the newborn vs. suckling comparison group (Figure 4), newborn hypermethylation genes include important nutritional metabolism-related genes (IGF2, PPARD). The suckling group is hypermethylated with genes related to fatty acid metabolism (LPIN1, ACOX2, ACOT7, GPD1), lipid and steroid metabolism (DHRS7B, SERPINA6, ERRFI1, NR0B1), glucose metabolism (HYI, GUSB, KCNQ1) and immune recognition, activation, and response (IL18, DCST1, B2M, TNFSF13, TNFSF13B, LILRB5). In the newborn vs. adult group, adult hypermethylated genes are related to cell proliferation (SDC2, MORF4L2), fatty acid metabolism (SCD, THRSP), and carbohydrate metabolism (GCK). Newborn hypermethylated genes are related to cellular processes (SKA1, BAIAP2, KLC1). In the suckling vs. adult group, the differences are centered on hypermethylated genes in the suckling group, such as immune correlation (PTPN7, CD68, C2) and lipid metabolism correlation (ACSM2A, PLIN2, HMGCS2).

## 4. Discussion

DNA methylation is a dynamic process that is influenced by both internal genetic programs and external environmental alterations. Previous studies have focused on DNA methylation changes during primordial germ cell development and embryogenesis [7,8,9], and there is little known about the dynamics of DNA methylation during postnatal development. Therefore, this study provided a 5mC methylation profile after birth, comparing DNA methylation and gene expression in pig liver tissues of newborn, suckling, and adult stages.

In this study, we collected liver samples from 0-day-old (newborn), 21-day-old (lactation), and 2-year-old (adult) Rongchang sows for BS-seq differential methylation analysis. The average data volume of six filtered samples was about 97 G, while the average data volume of liver samples in another study of weaned piglets was only 0.04 G. This was also due to the larger sequencing depth we chose. 

PCA analysis of CG methylation levels was performed on all liver samples, and this analysis showed a clear separation between samples from different periods. Through cluster analysis, we found that the adult and lactating groups clustered together and separated from the neonatal components, indicating significant differences between age groups in terms of CG methylation levels. In another study of human liver samples, differences in methylation levels were also found between fetal and adult groups [35]. By classifying the methylation sites, we found that the methylation of the result samples existed in CG, CHG and CHH (H = A, C or T), and most of the DNA methylation occurred at CG sites, which was consistent with the results of previous studies [36]. On this basis, we further explored the average CG methylation levels of promoter, utr5′, exon, intron, utr3′, CGI and CGI_shore repeats in liver samples at three stages, and found that the overall methylation levels of samples at different periods were similar, but that the average methylation levels of different components were significantly different. utr5′ had the lowest methylation level and utr3′ had the highest methylation level. A similar conclusion was found in another study of fetal bovine livers, where both low-nutrient and high-nutrient fetal livers had the lowest levels of methylation in utr5′, with significantly higher methylation levels in utr3′ and duplicate regions compared to other regions [37]. Further study showed that the methylation level was significantly reduced at 2 kb upstream of the gene start site, and reached the lowest in the region around the gene start site. Gene regions showed a sharp increase, followed by another decline after the gene end site, a pattern of methylation similar to that seen in multiple species [24,25,26,27,28]. Previous studies have shown that when the main source of nutritional requirements of newborn pigs changes from glucose in umbilical cord blood to lipids in breast milk, metabolic pathways such as gluconogenesis, fatty acid β oxidation and de novo lipogenesis are upregulated in the liver, and liver fatty acid β oxidation is gradually activated during the neonatal period, thus generating energy from breast milk lipids [38]. In this study, RNA-seq functional enrichment analysis showed that newborn pigs were specific in terms of fatty acid metabolism, indicating that the main nutritional requirements of newborn pigs were derived from fatty acids in breast milk. However, the expression of immune-related genes in the neonatal period is higher than that in the lactation period, and previous mouse studies also found that with the development of liver, the genes involved in the expression of immune cells gradually decreased [39]. Similarly, due to the transformation of food sources, the related metabolism, dominated by fatty acids, is transformed into multi-nutrient-related metabolism, in which energy metabolism is dominated by carbohydrates [40,41]. After adulthood, the metabolic diversity of the body is reduced, showing a strong lipid metabolism ability. In another study, it was also found that adult pigs had stopped growing and had lower energy requirements and metabolic rates compared to lactation. Adult pigs need more stable energy sources and are more inclined to use long-chain saturated fatty acids for long-term energy storage and utilization. Moreover, adult pigs have matured their digestive system and metabolism and can make more effective use of long-chain saturated fatty acids [42].

The pattern of the regulation of DNA methylation across the genome is complex and occurs in different gene regions. It is generally believed that DNA methylation in the promoter region is associated with decreased expression [4,5]. However, the correlation between certain methylation patterns within genes and gene expression is complex. Therefore, the detection and analysis of overall gene methylation levels and patterns in tissues at different growth and development stages are crucial for understanding the relationship between tissue spatio-temporal-specific methylation and tissue spatio-temporal-specific gene expression. In this study, through comprehensive analysis of DNA methylation and gene expression in the promoter region, it was found that there was a large number of DMRs whose methylation and expression levels were negatively correlated in liver samples from the three periods. We further identified DEGs whose methylation and expression levels were negatively correlated in promoter DMRs and found that, in the neonatal group, SLC44A3, SLC27A5, ARRDC3 and other genes were hypomethylated and highly expressed, and that the high expression of these genes was conducive to the metabolism of fat energy in colostrum in neonatal pigs. SLC27A5, which is involved in fatty acid transport and bile acid transport, has been shown to be associated with methylation and influence the development of non-alcoholic fatty liver disease [43], and a high level of expression of these genes helps new pigs to metabolize fatty energy in colostrum. We found that IGF2, a key factor in nutrient metabolism, was hypermethylated in the neonatal group. Studies have found that the methylation level of IGF2 after birth is related to obesity, and a reduction in IGF2 methylation will increase the risk of metabolic diseases, and this process is affected by methylation [43]. At the same time, we found the hypermethylated gene PPARD in newborns, suggesting that PPARD affects the function of the PPAR pathway through dietary activation, thereby activating the β-catenin pathway [44]. Compared with the lactating group, hypomethylized and highly expressed LPIN1 in the neonatal group enhanced hepatic mitochondrial fatty acid oxidation through PPRRα and PPARγ [45]. Since the growth of pigs is stagnant after adulthood, the energy demand and metabolic rate are lower, and thus there is a need for more stable energy, meaning so the synthesis and storage of long-chain saturated fatty acids are more inclined [42]. In the comparison between the weaning and adult groups, we found that the hypomethylation of the HMGCS2 gene with a high expression level in the adult group can promote the β-oxidation of fatty acids [46]. Meanwhile, PLIN2, which is associated with enhanced fat storage, was also found to be highly hypomethylated in the adult group. Compared with the neonatal group and the lactating group, the differentially expressed genes showed that the liver’s ability to metabolize sugar was gradually enhanced in the adult group, indicating the influence of food sources on metabolic ability and the regulation of related genes. Previous studies on mice have shown that food source has a strong influence on the expression of related genes [38].

The liver is also an important immune organ. The immune establishment of the liver after birth is extremely complex and is regulated by many factors. In this study, we found that TNFSF13B was highly expressed in the newborn group by hypomethylation. Studies have shown that human infant TNFSF13B is the most demethylated gene in the TNFSF family, with a 20% decrease in β after birth [47]; STAT2 plays an important role in the immune response to intracellular and extracellular stimuli, including in inflammation, foreign substance invasion, and cancer [48]. We found that STAT2 is hypermethylated in the lactating group and less methylated in the newborn and adult groups, but its expression levels are high. In addition, DCST1, which interacts with SATA2 to regulate the antiviral effects of type I interferon [49], was found to be hypomethylated and highly expressed in the neonatal group. AOX1 is also highly hypomethylated in the neonatal group and is involved in drug metabolism. Studies have shown that the degree of the methylation of its promoter has an impact on its expression [49]. IL18, a pro-inflammatory pleiotropic cytokine, is also highly expressed in both newborn and adult groups, and it has a key role in activating lymphocytes, NK cells, and T cells [50]. B2M was also highly expressed in the newborn group; this is involved in immune surveillance [51]. In the adult group, PTPN7 (associated with T-cell activation) [52], CD68 (involved in macrophage function) [53], and GIMAP1 (involved in B-cell activation) were highly expressed and hypomethylated [54]. These results indicate that DNA methylation is widely and significantly involved in liver immunity. 

We found significant differences in the expression and methylation levels of genes related to lipid metabolism in each stage, and similar results were found in the study of Rao et al. [42]. This was potentially related to the diet and nutritional composition of pigs at different developmental stages [38]. At the same time, DNA methylation was widely and significantly involved in liver immunity (Figure 3 and Figure S4). Compared with the lactation group, the newborn group and the adult group had higher expression levels of genes related to body immunity, but lower methylation degree. Studies have also shown that the early liver has a large number of innate and adaptive immune cells, ensuring that the fetus has a certain resistance at birth [55]. During lactation, piglets acquire immune protection through prolonged breastfeeding, resulting in a diminished demand for autoimmune gene expression. By the adult stage, the function of the immune system has stabilized and been perfected, with T cells and NK cells gradually becoming the main immune cell populations [42], so that the expression of immune genes is maintained at a high level.

In conclusion, we investigated DNA methylation levels in the livers of Rongchang pigs during neonatal, lactation, and adult stages of life. At the epigenome scale, we identified many genomic and promoter differential methylation genes related to nutrient metabolism and immunity. Compared with lactation and adulthood, a large number of genes related to fatty acid metabolism were hypomethylated and highly expressed in the neonatal group. Immune-related genes showed relatively high methylation levels and low expression patterns during lactation. This reflects the developmental maturation of epigenetic regulation during tissue development after birth. Some genes related to fatty acid metabolism were hypomethylated and highly expressed in the neonatal period, which was consistent with the high nutrient utilization capacity of breast milk in Rongchang pigs in the early stage, while the adult group was more inclined to store and utilize fatty acids, especially long-chain fatty acids. At the same time, the liver is a reservoir of immune cells waiting for activation by external factors after birth, but the downregulation of immune-related genes during lactation suggests the importance of disease resistance at this stage, and its regulatory mechanism needs to be further studied. 

## Data Availability

The BS-seq data have been deposited into the NCBI Gene Expression Omnibus (GEO) under the accession GSE266050.

## Supplementary Materials

The following supporting information can be downloaded at: https://www.mdpi.com/article/10.3390/genes15081067/s1, Table S1: Quality control statistics of raw data; Table S2: Statistics of reads mapping to reference genomes; Table S3: Summary of genome coverage statistics; Table S4: Functional enrichment analysis results of DEGs from RNA-seq data; Table S5: Results of promoter DMRs with the gene expression significant test; Table S6: Functional enrichment analysis results of genes with hypermethylated promoters; Table S7: Genes with higher DNA methylation level and lower expression; Table S8: Functions of hypermethylated genes with lower expression; Figure S1: Clustering of liver samples based on methylation level of 2 kb windows of the whole genome CG site; Figure S2: Analysis of differentially expressed genes (DEGs) between stages; Figure S3: The KEGG pathway with gene-promter differentially methylated genes between stages in the liver. Figure S4: Differences in expression and methylation levels of specific genes at different stages.

## Abbreviations

BS-seq, bisulfite sequencing; DMRs, differentially methylated regions; CpG/CG, cytosine–guanine; 5mC, 5—methylcytosine; GEO, gene expression omnibus; DEGs, differentially expressed genes; TSS, transcription start site; CGI, CpG islands; TES, transcription stop site; PCA, principal component analysis; GO, gene ontology; KEGG, Kyoto encyclopedia of genes and genomes; GO-BPs, GO biological processes; hyper, hypermethylated; hypo, hypomethylated.
