# Supplementary figures and images for: DNA Methylation of Postnatal Liver Development in Pigs
Source: Genes (Basel). 2024 Aug 13;15(8):1067. doi: 10.3390/genes15081067 (PMC11353940; doi:10.3390/genes15081067)

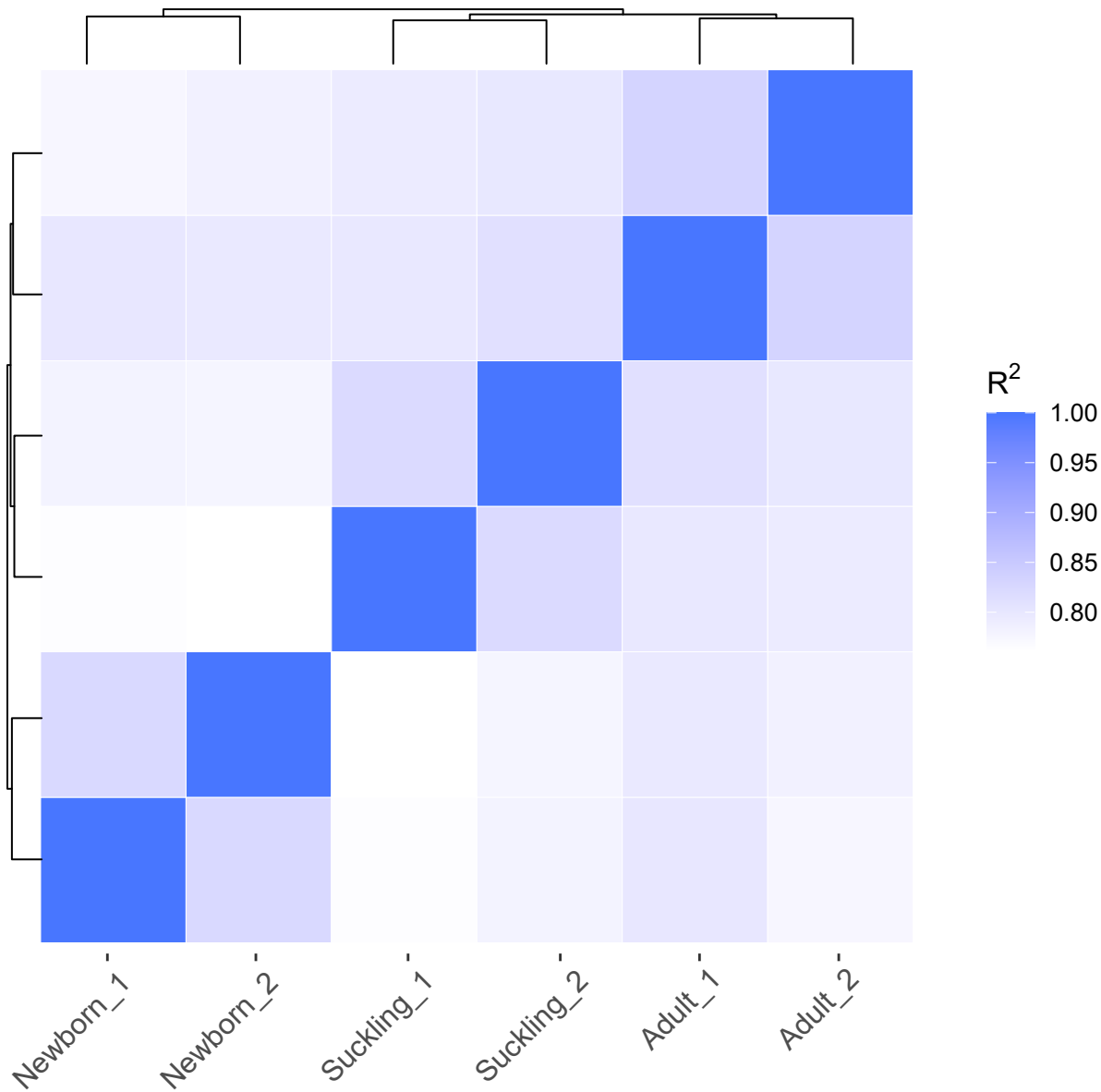

Supplement: Supplementary file 1 [file genes-15-01067-s001.zip › Figure S1.pdf]

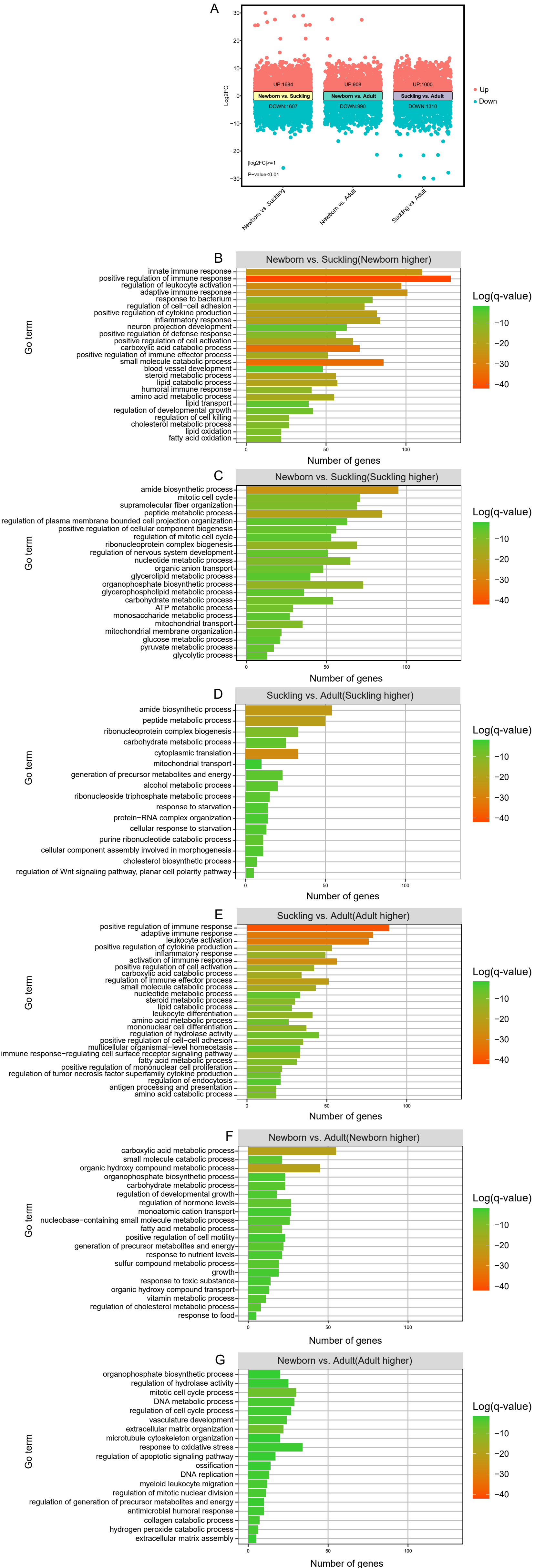

Supplement: Supplementary file 1 [file genes-15-01067-s001.zip › Figure S2.pdf]

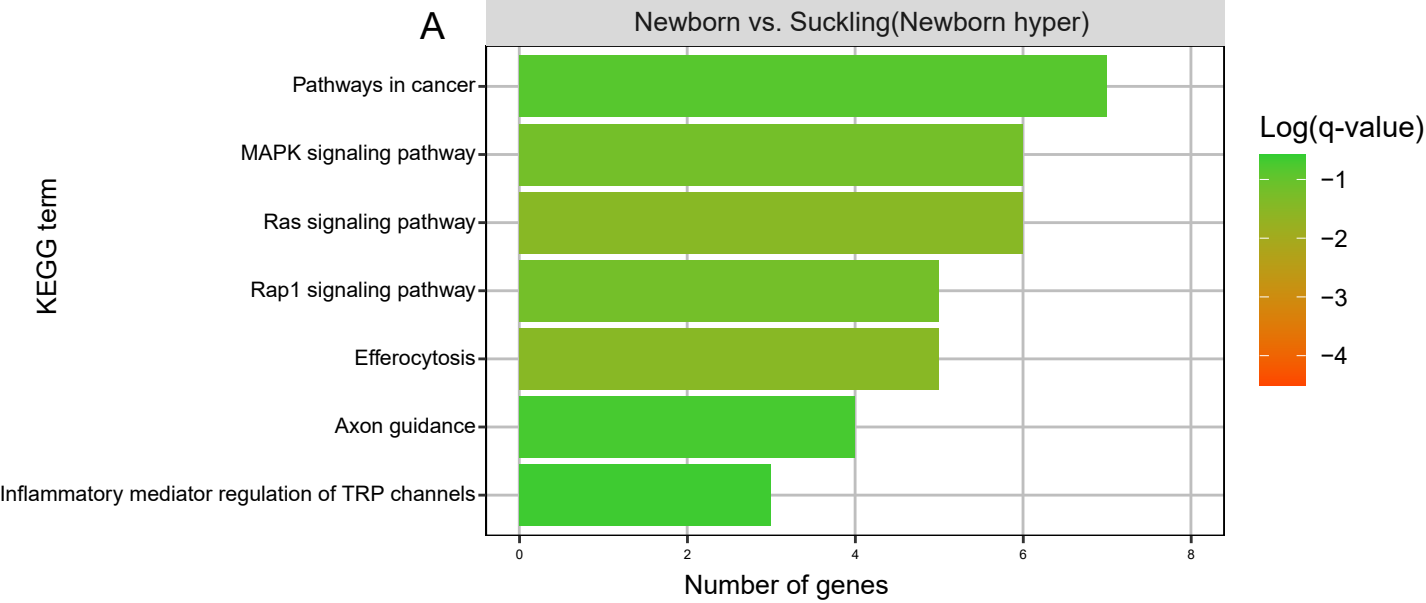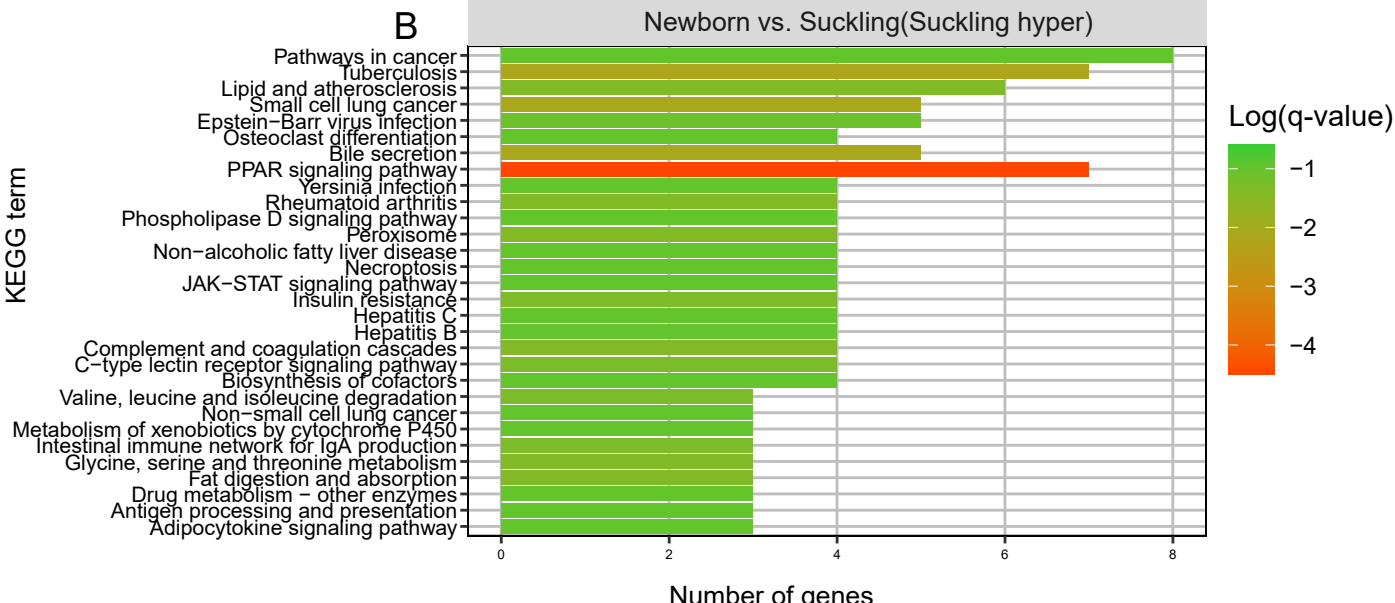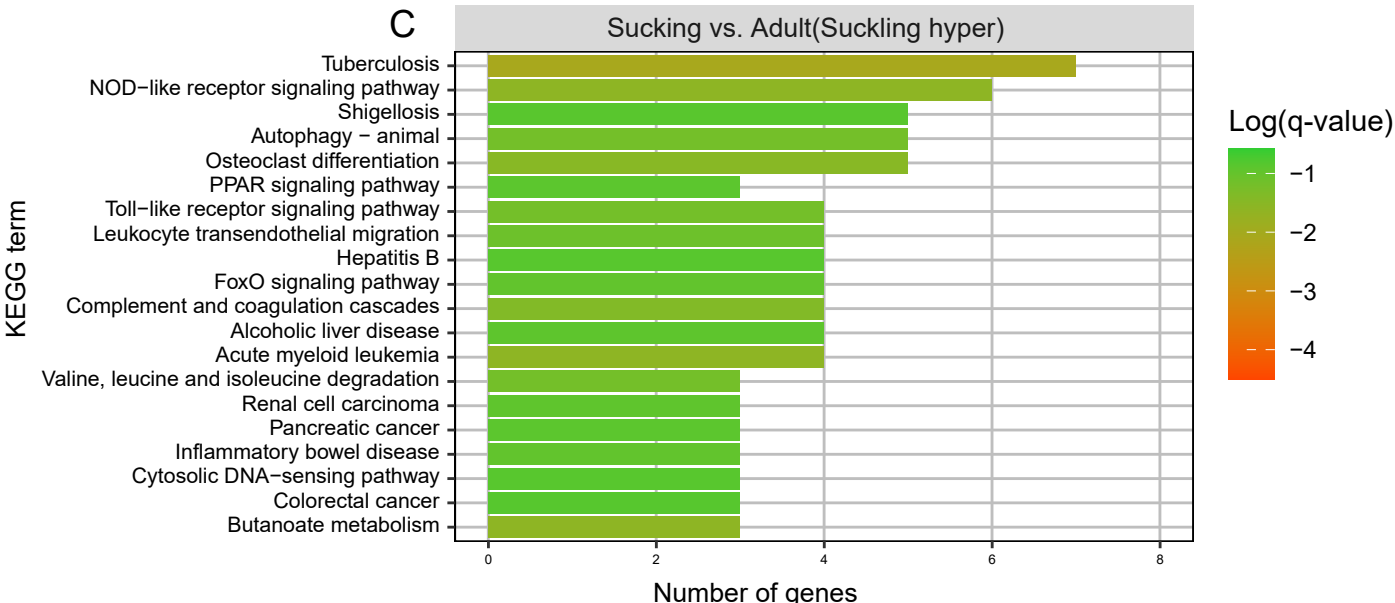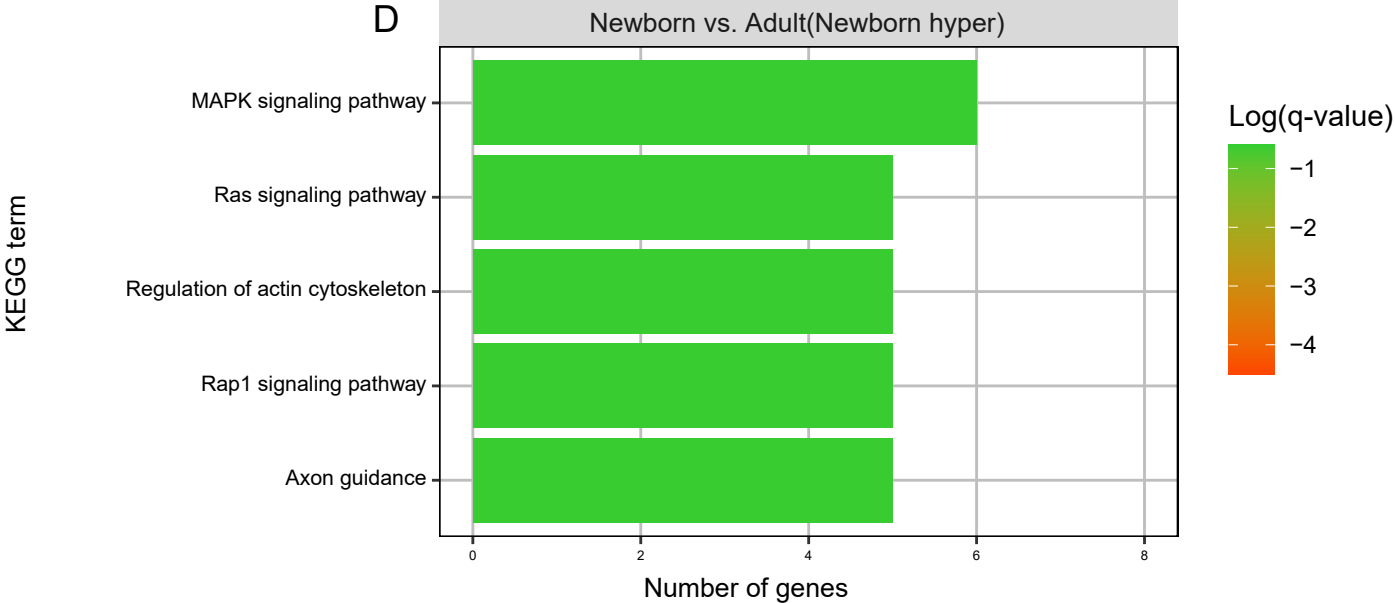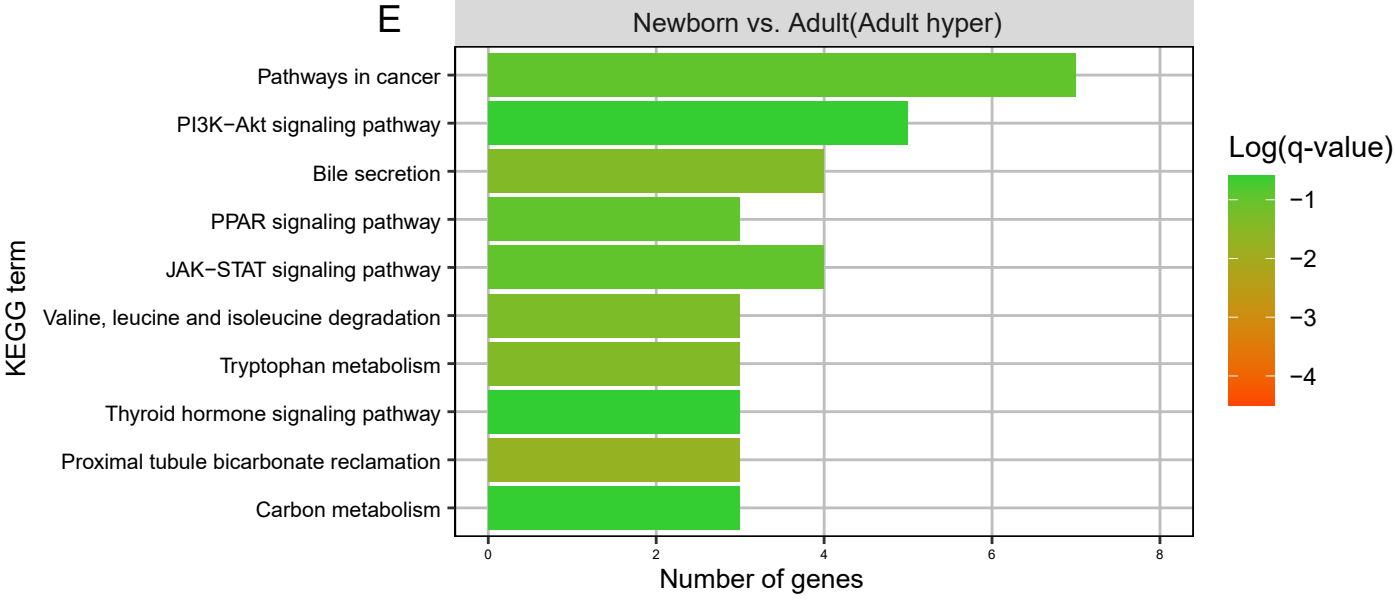

Supplement: Supplementary file 1 [file genes-15-01067-s001.zip › Figure S3.pdf]

A

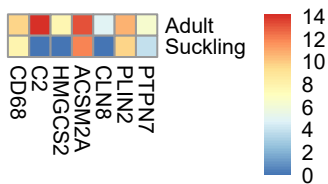

B

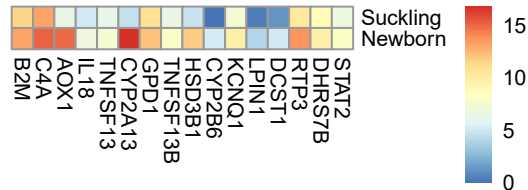

C

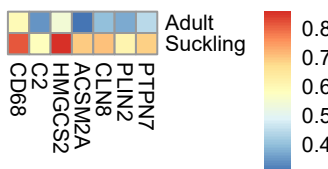

D

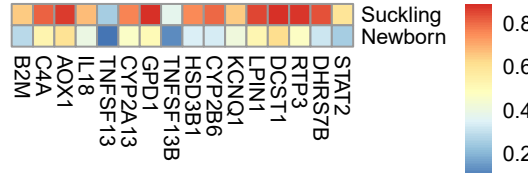

E

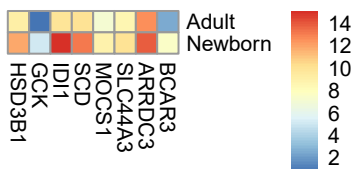

F

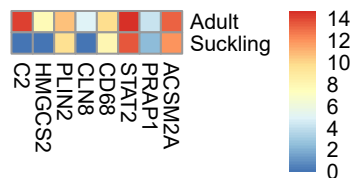

G

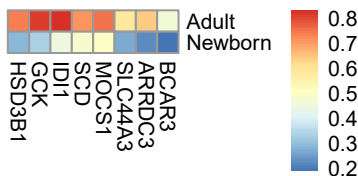

H

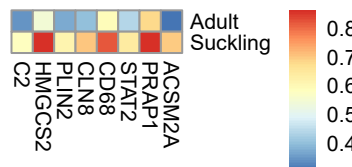

I

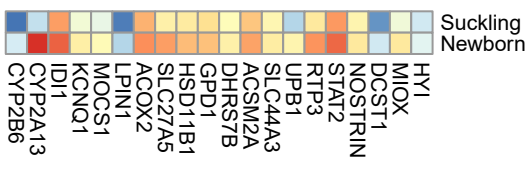

J

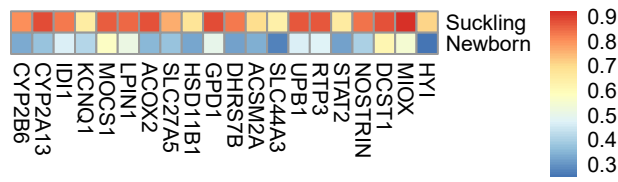

Supplement: Supplementary file 1 [file genes-15-01067-s001.zip › Figure S4.pdf]
